# An iterative block-shifting approach to retention time alignment that preserves the shape and area of gas chromatography-mass spectrometry peaks

**DOI:** 10.1186/1471-2105-9-S9-S15

**Published:** 2008-08-12

**Authors:** Minho Chae, Robert J Shmookler Reis, John J Thaden

**Affiliations:** 1 UALR/UAMS Joint Graduate Program in Bioinformatics, University of Arkansas at Little Rock, Little Rock, AR 72204, USA; 2 Department of Geriatrics, University of Arkansas for Medical Sciences, Little Rock, AR 72205, USA; 3 Department of Biochemistry and Molecular Biology, University of Arkansas for Medical Sciences, Little Rock, AR 72205, USA; 4 Central Arkansas Veterans Healthcare System LRVA-151, 4300 W. 7^th ^Street, Little Rock, AR 72205, USA

## Abstract

**Background:**

Metabolomics, petroleum and biodiesel chemistry, biomarker discovery, and other fields which rely on high-resolution profiling of complex chemical mixtures generate datasets which contain millions of detector intensity readings, each uniquely addressed along dimensions of *time *(*e.g.*, *retention time *of chemicals on a chromatographic column), a *spectral value *(*e.g., mass-to-charge ratio *of ions derived from chemicals), and the *analytical run number*. They also must rely on data preprocessing techniques. In particular, inter-run variance in the retention time of chemical species poses a significant hurdle that must be cleared before feature extraction, data reduction, and knowledge discovery can ensue. *Alignment methods*, for calibrating retention reportedly (and in our experience) can misalign matching chemicals, falsely align distinct ones, be unduly sensitive to chosen values of input parameters, and result in distortions of peak shape and area.

**Results:**

We present an iterative block-shifting approach for retention-time calibration that detects chromatographic features and qualifies them by retention time, spectrum, and the effect of their inclusion on the quality of alignment itself. Mass chromatograms are aligned pairwise to one selected as a reference. In tests using a 45-run GC-MS experiment, block-shifting reduced the absolute deviation of retention by greater than 30-fold. It compared favourably to COW and XCMS with respect to alignment, and was markedly superior in preservation of peak area.

**Conclusion:**

Iterative block-shifting is an attractive method to align GC-MS mass chromatograms that is also generalizable to other two-dimensional techniques such as HPLC-MS.

## Background

Originally employed to analyze single or a small collection of targeted molecules, gas chromatography-mass spectrometry (GC-MS) and other chromatography-spectrometry technologies have emerged as viable tools for the wholesale fingerprinting of complex chemical mixtures. This has been made possible by the advent of computer-aided chemometrics, which in principle can lead to identification and quantification of most or all component chemicals. This advancement continues to profoundly benefit scientific disciplines as diverse as petroleum, diesel and biodiesel chemistry [1,2]; biomarker discovery [3]; basic metabolic chemistry; drug metabolite identification; receptor-ligand and enzyme-substrate biochemistry; environmental toxicology [4]; pharmacokinetics; functional genomics [5] and metabolomics [6,7].

**Figure 1:**
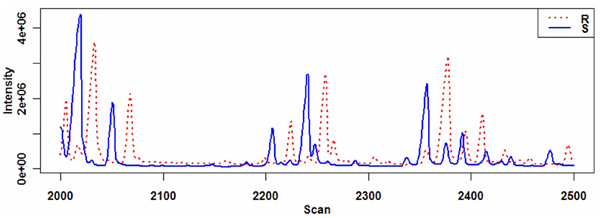
**Unaligned chromatograms**. A 500-scan region is shown for each of two total-ion-current chromatograms, *S *and *R*, in a GC-MS experiment.

Separations with mass detection yield *mass chromatograms*; with the intensity of the mass detector's response indexed both to the ion mass-to-charge ratio (*mz*) channel being monitored, and to the time elapsed since injection of the biochemical mixture onto the chromatographic column positioned upstream of the detector, *i.e*., to its retention time (*RT*). With modern mass spectrometers, the variation in *mz *of a chemical is usually modest and often can be ignored during data processing. *RT *variation can be appreciable, however, as illustrated in Figure 1, and nonlinear over the extent of a chromatogram as dramatically illustrated in Smith et al. [8] and elsewhere [9,10]. Retention-time differences are caused by uncontrolled experimental variables such as column aging and instabilities in flow rates of mobile phases and the shape of thermal or mobile-phase gradients [9,11,12]. Misalignment was a minor issue as long as multidimensional separation technologies were used to quantify a few molecular targets, but manual curation proves arduous if not impossible when each mass chromatogram displays hundreds of potentially significant features and an experiment contains hundreds of such analytical runs.

The data produced from chromatography coupled with mass spectrometry can be viewed as *three-way*: along *RT *space, *mz *space, and analytical run space. Some of the most attractive and powerful three-way techniques, such as parallel factor analysis (PARAFAC) to further resolve peaks, assume *trilinearity *of data, a mathematical constraint such that multiple instances of a data feature align with each other along all three dimensions, which rarely if ever is achieved in real mass-chromatographic data, primarily due to *RT *misalignment. Techniques making no trilinearity assumption (*e.g.*, PARAFAC2 and MCR-ALS) still usually require alignment to facilitate parsing of the large matrix representing an entire, typical chemical profiling experiment into submatrices of computationally feasible size. This is particularly true since parsing must occur at locations along the chromatogram lacking peaks. Finally, two-way, one-run-at-a-time approaches such as AMDIS [13], when applied serially to multiple runs, *e.g.*, with the help of MET-IDEA [14] or SpectConnect [15], have been observed to produce an artifact where single chemical eluates are identified as multiple mass-chromatographic features [16], again largely the result of misalignment. Thus, a complete comparative analysis of data acquired in a non-targeted, profile-type experiment, involving many analytical runs, needs to include a robust alignment operation as an obligatory preprocessing step.

Alignment algorithms have been described as falling within two categories based on whether they use feature detection or not [10]. The best-known method that does not detect features is correlation optimized warping (COW), proposed by Nielson et al. [17]; it warps, i.e., linearly interpolates, one chromatogram to another by selecting input parameters such as section length and slack size that maximize the similarity between the two chromatograms using dynamic programming. Optimization of the input parameters is difficult, however, and performance is often questionable [18,19]. Variant warping algorithms, such as parametric and semi-parametric time warping, have been proposed to address these deficiencies (reviewed in [19]). Feature-detection algorithms, in contrast, attempt to identify and match peaks throughout an entire set of runs. Although this approach requires one additional step for alignment, it generally produces superior results and adds the ability to integrate peak areas during the process. Recent examples of such methods include metAlign, MZmine, and XCMS [8,20,21]. These methods differ with regard to which features are used for matching, some employing only features evident in *RT *space [2,22], while others also use spectral information [8,10,11,20].

We have developed and tested an improved *RT *alignment method that relies on feature detection and utilizes matching criteria based on both peak retention time and peak spectral data. Peaks in sample mass chromatograms are detected and matched to peaks in an arbitrarily selected reference chromatogram. Mass spectra provide information required to determine whether peaks from different samples are chemically identical components. In addition to retention data and mass spectra, our method utilizes an inherent property of chromatograms: peaks eluting near to each other tend to show similar deviations in their retention times, and thus can be initially processed as blocks of peaks. Through trial, or simulated, shifts of blocks along the *RT *axis relative to the reference chromatogram, and through reorganization of peaks into new blocks as needed, an optimal shift strategy is discovered. This shift information is applied to both the TIC and the full, two-dimensional matrix of raw data while warping only non-peak regions, in an effort to exactly preserve the shapes and integrated areas of key peaks. Thus, the result matrix can be used as a direct input to subsequent multivariate analysis.

## Results

### Algorithm

Our alignment method operates in a pairwise fashion: one mass chromatogram, a sample *S*, is aligned with a reference chromatogram, *R*. *R *can be any run from the set of all runs but, once selected, must be used for the entire set. Chromatographic peaks with acceptable signal-to-noise ratio (*SN*) and width are detected in *R*, and for each *S *as its processing is begun, by analyzing chromatograms with a published wavelet-based method [23]. A *peak set *contains only those peaks actually used in the alignment process, accompanied by further information about them. An *S *peak set *SP *always includes all detected peaks that satisfy *signal-to-noise *and width criteria. An *R *peak set *RP *will typically contain only a subset of all peaks; it is a dynamic set, in that it is selected anew for every *S*, using only peaks most compatible with the *S *being processed. Accurate alignment is possible through the matching of peaks detected in select *mz *channels, *i.e.*, in select extracted ion chromatograms (EIC), instead of in the TIC, where coelution and higher baselines can muddy the picture. Overall, the alignment process for a pair of chromatograms involves (a) finding EIC peaks for the two, (b) iteratively matching them, which also yields retention discrepancy data, and (c) aligning, *i.e.*, warping and shifting, the sample chromatogram based on peak-match data. This pairwise alignment is repeated for every sample, matching to the same reference. Only the first two steps will be explained in detail in this paper.

### Peak detection

**Figure 2:**
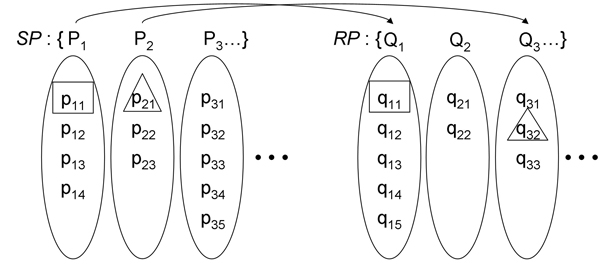
**Diagram of two peak sets**. Peaks in a sample peak set *SP *match with peaks in a reference peak set *RP*. *SP *is composed of detected TIC peaks, *i.e*, capital *P*'s, in which individual EIC peaks, *p*'s, up to five, are arranged in descending order of their signal-to-noise ratios. Note that *RP *is a peak set composed of "inferred TIC" peaks since individual EIC peaks, *q*'s, are first identified by using the *mz *values of *SP *and then are grouped into a TIC peak. This also illustrates the matching of TIC peak *P*_1 _to *Q*_1_, but of P_2 _to *Q*_3 _because, in the latter case, either peak *Q*_2 _had no EIC component (*q*_21_*and q*_22_) with a matching *mz *value, or the spectra of *P*_2 _and *Q*_2 _were insufficiently correlated, whereas Q_3 _met both of these conditions, with *q*_32 _having matching *mz*.

The purpose of this step is to assemble two sets of peaks, for *S *and *R*, such that they closely resemble each other in size and in the mass-spectral characteristics of their elements. Let the set of peaks from a sample TIC be *SP *= {P_1_, P_2_,..., P_n_} and from a reference "inferred TIC" (details below) be *RP *= {Q_1_, Q_2_,..., Q_m_}. Their elements are ordered by elution time and each element can be envisioned as a group of EIC peaks, one resulting from each ion produced upon ionization, with possible fragmentation, of an eluted chemical component. Up to five EIC peaks per TIC peak are recorded in descending order by their signal-to-noise ratio (SN), thus, for instance, element *x *of *SP *is the set of EIC peaks, P_*x *_= {p_*x*1_, p_*x*2_,... p_*xj*_} where 1 ≤ *x *≤ *n *and 1 ≤ *j *≤ 5. Similarly for *RP*, Q_*x *_= {q_*x*1_, q_*x*2_,..., q_*xk*_}, 1 ≤ *x *≤ *m*, 1 ≤ *k *≤ 5, as illustrated in Figure 2. In this peak detection stage, all necessary EIC peaks are found for the alignment, accompanied by the requisite peak information: *mz*; width; retention time of apices; and *SN*. Since values for the time axis are in units of MS scan number and are discrete values, perhaps as few as eight across a peak, the peak maxima found by a peak detection algorithm will often deviate from their true apices. Thus, for more precise alignment, fractional top positions are determined for use in the actual alignment. It should be noted, however, that subsequent alignment involves only integral shifts in scan number, in order to preserve the matrix-like structure of mass-chromatographic data. Considering the three points acquired nearest the apex of a peak, each an ordered pair (x, y) where x is scan and y is intensity, we can solve the quadratic equation *y *= *Ax*^2 ^+ *Bx *+ *C *describing the unique downward-opening parabola defined by those points, using simple linear algebra. The true apex occurs at the position where the first derivative is zero, and will equal (-*B/2A*, *C-B*^2^/*4A*).

For actual detection of both TIC and EIC peaks, we use the continuous wavelet transform algorithm of Du *et al*. [23] since it is robust to noise and readily available in the authors' MassSpecWavelet package for the R statistical language [24]. After NetCDF [25] files of *S *and *R *are read into matrices of intensities, the peak detection method proceeds as follows (symbol conventions are summarized in Table 1):

**Table 1:** Algorithm input variables

**Variable**	**Formal Definition**	**Default**
*Sntic*	*SN *threshold when detecting peaks in *S *TIC. Low values are used in order to include weak signals.	1
*Sneic*	*SN *threshold when detecting peaks in EIC chromatograms for *S *and *R*. Values higher than the *SNtic *are used to reduce the risk of matching noisy EIC peaks.	5
*PWidth*	Peak width threshold for every peak detection, in units of scans.	12
*PClose*	EIC-to-TIC peak apex distance threshold, in units of scans.	2
*SDist*	Search distance when finding candidate peaks in *R*, in units of scan number measuring from the apex of an *S *peak.	15
*CorMass*	Correlation coefficient threshold between two peaks.	0.95
*Prof*	Profile threshold for peak deviations.	0.5
*LpBound*	Lone peak boundary, in units of scan number.	5

1. Detect peaks in a sample TIC *S *whose *SN *ratios are greater than *SNtic*.

2. Form peak set *SP *by finding, for each detected TIC peak, as many component EIC peaks as possible, not to exceed five, for which the distance between its apex and that of the TIC is less than *pClose*, the *SN *is greater than *SNeic*, the peak width is less than *pWidth*; and the *SN *is among the five highest *SN *of all EIC peaks passing these criteria. Retention times are expressed in fractions of scans by a quadratic interpolation of their apex positions, as described above.

3. For each peak in *SP*, find EIC peaks in the reference run *R*, which have corresponding *mz *values.

4. Group the found EIC peaks into a peak set *RP*, an "inferred TIC" peak set, by requiring that their apices fall within *sDist *of the corresponding EIC peak in *S *and within *pClose *of the inferred TIC peak in *R*. Additionally, the Pearson correlation of two numeric vectors of *mz*-ordered ion intensities, *i.e.*, spectra for the *S *TIC peak and the *R *inferred TIC peak, whose location is taken as the median *RT *of the grouped EIC peaks, must be greater than *corMass*.

### Iterative peak matching

**Figure 3:**
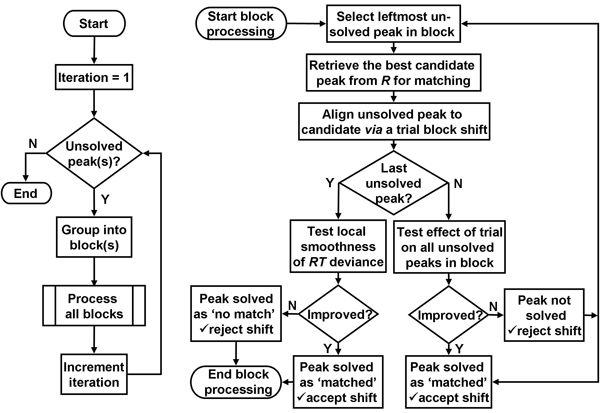
**A flowchart of iterative peak matching**. The left flowchart shows the overall iterative peak-matching flow, whereas, the right flowchart shows the flow within the subroutine for processing a single block.

Once the peak sets for *S *and *R *are determined, peak matching can be initiated. Figure 3 illustrates the overall process by which all *S *peaks are *solved*, *i.e.*, matched to an *R *peak or determined to have no match. The basic unit for iterative peak matching is a *block *which is composed of adjacent, unsolved peaks in *S *(or a single one); blocks are bound by either solved peaks or ends of the chromatogram. Initially, in the first iteration, the entire *S *peak set is one block. Each iteration identifies those peaks which remain unmatched, organizes them into new blocks, identifies new *S-to-R *peak matches within blocks, and discovers an *RT *shift value for each match that optimizes the alignment of subsequent peaks in its block. Besides recording the match, the method records the iteration number when a match was made, EIC *mz *information, and, importantly, the retention discrepancy, *i.e.*, the nearest integral number of MS scans by which the sample peak will need to be shifted in the final warp-and-shift alignment step to align it with the corresponding reference peak. After the final iteration, no peak in *SP *remains unsolved, *i.e.*, all are either matched with a reference peak, or evaluated as being unmatchable. In any iteration, if a peak is solved, its final location is fixed and no further adjustment will be made to it in later iterations.

Retrieval of the best candidate peak from *R *against which to test the current *S *peak (Figure 3, upper right) is illustrated also in Figure 2 (squares and triangles). The first step, comparing TIC peaks, ideally results in pairings of chemically identical chromatographic eluates. For this, one must exploit their underlying mass spectra. Spectra are treated as vectors of mass intensities and tested against each other by requiring that their Pearson correlation exceed a certain value. Once this criterion is met, the prominent EIC components of their spectra are tested to find the *mz*-matched EIC pair with the strongest *SN*. These are the "model" EIC peaks for that TIC peak pair, and their peak retention times are used instead of TIC retentions for more precise shifting.

The peak matching method produces a set of matched results. A peak match is represented by a list containing an *S *EIC peak, the matching *R *EIC peak, the *mz*, the shift amount, and the final iteration number, *e.g.*,

(1) {(p_11_, q_11_, 30, 5, 1), (p_21_, q_32_, 40, 3, 1), (p_31_, ϕ, 40, 3, 2),...}

where ϕ means there is no matching peak in *R*. Peaks are processed one-by-one according to their elution times. The current peak is matched only when (i) a candidate peak in *R *is within *sDist *of it, (ii) the Pearson correlation of the two peaks' mass spectra is greater than *corMass*, and (iii) the profile value of remaining peak deviations is greater than *prof*.

A profile value is determined as follows: for a block of peaks *SB *= {b_1_, b_2_,..., b_*l*_}, where b_1 _is the current working peak and *l *does not exceed the initial size of *SP*, initial deviations of peaks from their candidate matches can be represented by the vector *ID *= {id_1_, id_2_,..., id_*m*_}, where *m *≤ *l*. The alignment that would perfectly align b_1 _is simulated by shifting all the peaks in *SB *by the integer-rounded value of id_1_, resulting in a vector of deviations after the simulation, *SD *= {sd_1_, sd_2_,...}; note that sd_1 _is less than |0.5|. Next, we will have an evaluation vector E = {|id_1_| - |sd_1_|, |id_2_| - |sd_2_|,...} where absolute values of the simulated deviations are subtracted from absolute values of the initial deviations. A positive value within *E *means that its corresponding peak in *S *is brought closer as a result of the simulation. The, *profile value *is defined as the ratio of positive values to the total number of values in *E*. A profile value of 0.5 would mean that, if all peaks in a block were shifted by the initially recorded deviation of the current peak from its candidate peak in *R*, then half of the remaining traceable peaks, including the current one, are also improved in alignment. Only if the above three conditions (i, ii and iii) are met will the current peak be recorded as a match. Otherwise, it remains unsolved so that it can be processed again in later iterations with smaller block sizes. For the last peak in *SB*, however, there is only one element in the *E *vector, the current peak itself, so the profile value is always 1 and thus uninformative. In such a case, we model the deviations of already matched peaks by loess regression and use the model to predict the deviation of the current peak. If the actual deviation falls within *lpBound *of the prediction, then the candidate matching peak in *R *is accepted as a match.

**Figure 4:**
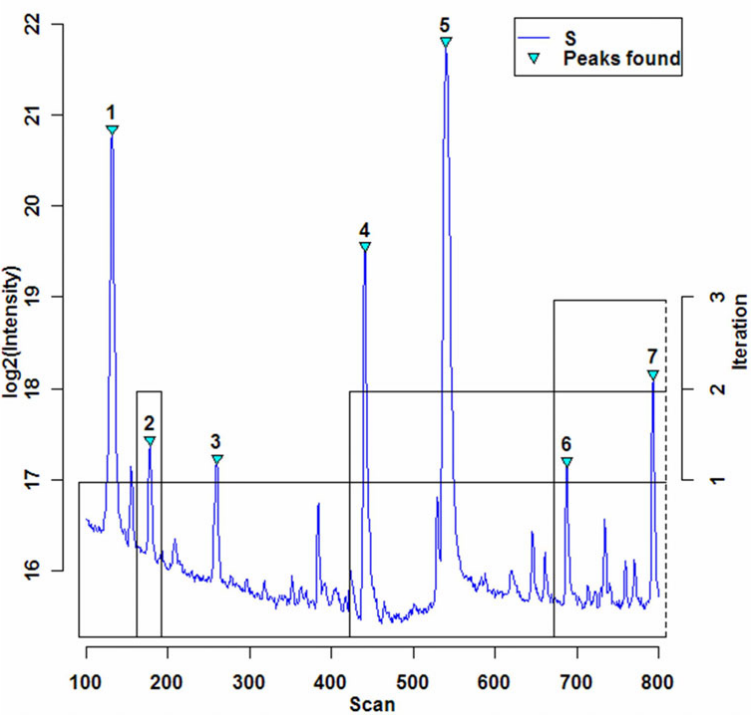
**An example of iterative peak matching**. A 700-scan region of *S *TIC is shown which has 7 detected peaks. Peaks are assigned to blocks at the start of each iteration, with blocks shown as boxes of height matching the iteration number. Intensities are log transformed for a better display of weak signals.

In such a case of a single or the last peak in a block, block processing will always solve the peak, either as a match or as unmatchable, signified by ϕ. After the last peak in *SB *is solved and no more blocks remain to be processed, the iteration number is incremented, unsolved peaks are grouped into new blocks, and the match process continues until there are no unsolved peaks. The final size of the time axis is actually determined by the result of the first iteration during which all peaks are in one block. Peak matching simulations in subsequent iterations can affect the time domain only within the boundary of the peaks within blocks. Iterative peak matching is described in Figure 4 and Table 2. Figure 4 illustrates peak matching in a 700-scan region containing seven peaks (numbered 1–7) used for alignment testing. After three iterations, all peaks were solved, i.e., matched or unmatched. Four boxes show peak blocks created at the start of an iteration. The *y *values 1, 2 and 3 shown on an axis on the right side of the figure indicate within which iteration corresponding peak blocks were processed. Table 2 shows shift amounts applied to the peaks in a block at the end of each iteration in the example of Figure 4. The processing of peaks in a block proceeds from left to right. A parenthesized shift number implies matching and ϕ means that a peak was determined to have no match. When a match occurs, that shift amount is propagated to the subsequent peaks in the same block. For instance, peak #5 was shifted 11 scans when peak 1 was matched, and an additional 4 scans for a match of peak #3. Since it was not matched in the first iteration, peak matching continues in the next iteration. When peak 4 was matched, a shift of -2 was propagated to peak 5 and, with the resulting total shift of 13, peak 5 was determined by criteria described in Figure 3 to be matched, thus ending its processing.

**Table 2:** Record of shifts during the peak matching stage in Figure 4.

peak	shift (in scan)			
	**i = 1**	**i = 2**	**i = 3**	**Total**
1	(11)	-	-	11
2	11	ϕ	-	11
3	(15)	-	-	15
4	15	(-2)	-	13
5	15	(-2)	-	13
6	15	-2	(-1)	12
7	15	-2	(-1)	12

### Testing

**Figure 5:**
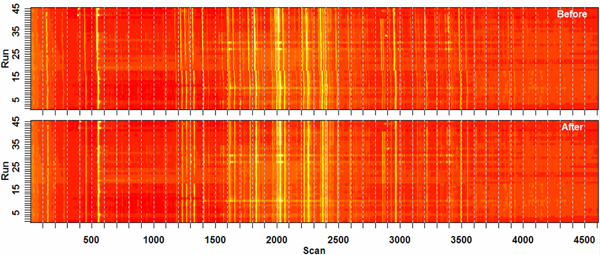
**Top plots showing all 45 runs, before and after alignment**. These two heat-map-encoded top plots display the total ion current (TIC) for mass chromatograms of all 45 runs in a *C*. *elegans *experiment (see text), before and after alignment. Run #8 was used as the alignment reference. The brightness is proportional to the logarithm of intensity, so peaks are displayed as bright vertical bars. Initially, as the run number increases, the peaks are skewed to left, meaning the same peaks eluted earlier in higher-numbered (later) runs. The pattern also exhibits serious breaks and other nonlinearities. These imperfections were corrected by the alignment method and are not evident in the bottom image.

Data in 45 files were used to test the alignment algorithm. They were acquired by a quadrupole GC-EI-MS system during a month-long study of the effect of life-span-altering mutations on metabolite levels in the soil nematode *C. elegans*. Unless specified otherwise, run #8 was selected as the reference and the rest of the runs were aligned to it in succession. Figure 5 shows TIC for all samples, viewed from above with total ion intensities color-encoded, before and after alignment.

**Figure 6:**
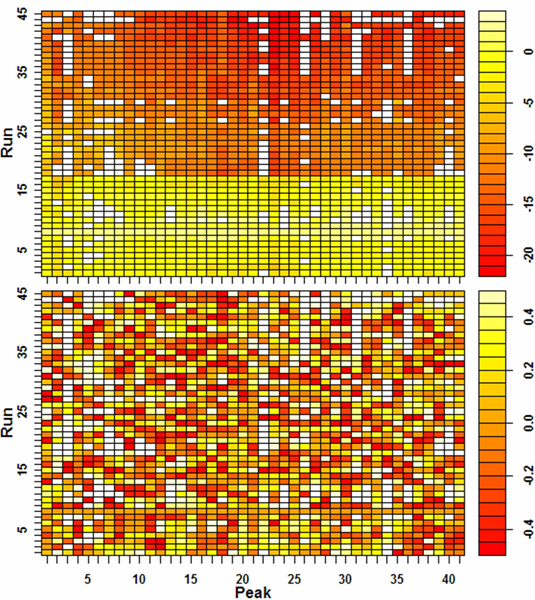
**Peak deviations before and after alignment**. Retention-time deviations of matched peaks are color-coded in these two panels, before (top) and after (bottom) application of the described alignment method. The heat-map code is displayed to the right (Note the narrower range of deviations represented by colors in the lower panel). White cells represent instances where a peak in a run either was not detected or did not pass signal-to-ratio and peak-width criteria. Twenty-one peaks were omitted from the display because they met criteria in fewer than ten sample runs; their inclusion does not alter the result. Run #8 again was selected as the alignment reference. Several weeks, and a GC-MS re-tuning operation, occurred between runs 17 and 18.

As shown in Figure 6, iterative block-shifting identified peak deviations for all runs and aligned them appropriately, thus, drastically decreasing peak deviations to no more that 1 scan, or an average deviation of 0.25 scans. Since a scan lasts 0.78 seconds, this is a mean deviation of 0.2 sec and a maximum deviation of less than one second for runs lasting over an hour. This is a great improvement over the initial deviations (as much as 22 scans or 17.2 seconds) and was achieved with conservation of the shapes and areas of key peaks, because only non-peak regions are warped.

The small remaining deviation results from the discrete nature of the chromatography time dimension.

**Figure 7:**
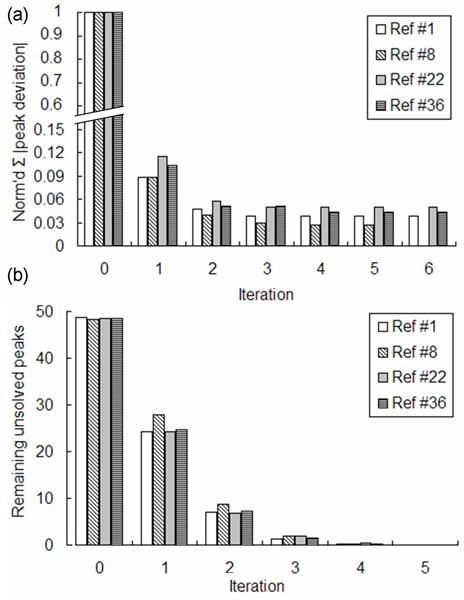
**Robust iterative peak matching with different references**. (a) Sums of absolute values of all sample-*vs.*-reference peak discrepancies are normalized to the pre-alignment (iter = 0) value and plotted as a function of the number of iterations completed. Data are compared for four independent alignments, with different runs selected as the reference. Regardless of which run was used as the reference, most peak retentions were corrected in early iterations (in the Ref #8 case, all peaks were solved by iteration 5 and no 6^th ^iteration was done). Comparing the same references, the number remaining unsolved after each iteration is shown in (b).

When the alignment was repeated on the same data but with different references, results were similar. Not only were similarly aligned chromatograms produced (see Additional Files 1, 2 and 3), but similar progress was made in correcting deviations and solving unsolved peaks as iterations progressed (Figure 7). No matter which reference was used, most deviations were corrected in early iterations.

### Comparisons

Two well known algorithms, COW and XCMS, mentioned earlier in this paper, were selected to further evaluate the performance of our block-shift method with respect to the correctness of the alignment and the preservation of peak areas, using the test data set. A full evaluation of the performance of the three methods under more diverse conditions could be the subject of a separate study, however, to our knowledge, even the limited comparison reported here between COW and XCMS is unprecedented. COW is available as a set of MATLAB scripts [26]; XCMS as an R package [8]. As in block shifting, analytical run #8 was chosen as the reference for COW. XCMS does not require the choice of a reference, relying instead on median positions identified, well-behaved *peak-groups *[8]. Four major TIC peaks were selected for these comparisons: one in the beginning; one near the end; and two from the middle of the time interval of chromatography. For each, the most prominent spectral *mz *value was identified, and its EIC chromatogram along the full extent of the chromatogram was used as the input for COW alignment. Both XMCS and block-shifting used as their input the entire set of EIC mass chromatograms for every run.

**Figure 8:**
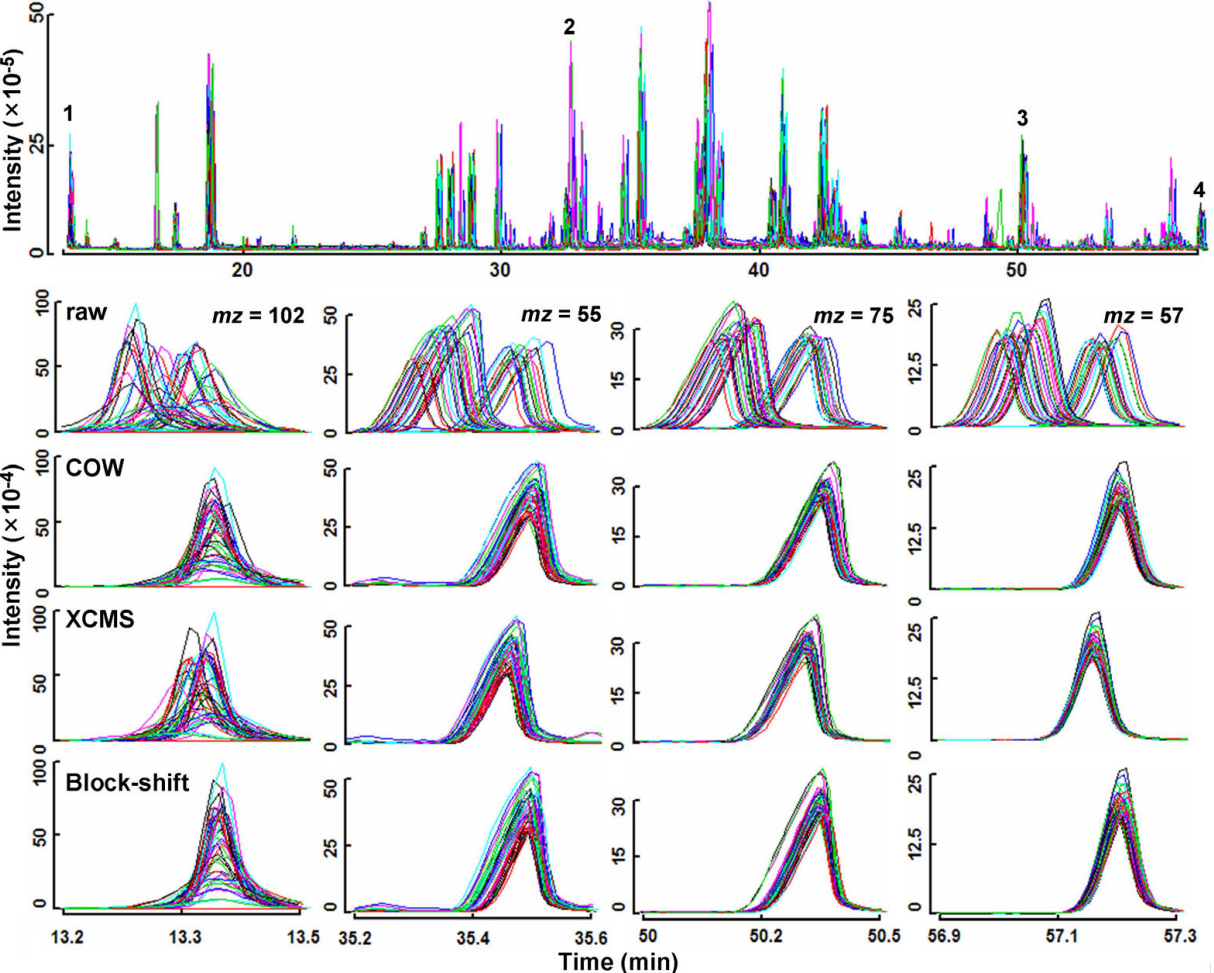
**A comparison of retention-time alignment by three methods**. Top panel: Unabridged total-ion-current (TIC) chromatograms for 45 analytical runs in a GC-MS metabolomics experiment, prior to alignment. Remaining panels: columns 1–4 show details for peaks labelled 1–4 in the top panel, both unaligned (top row), and aligned using COW [17] with automated parameter selection [26], using XCMS with three iterations [8], and using iterative block-shifting with its default parameters, as described in the text (rows 2–4, respectively).

The quality of alignment by these three approaches is compared in Figure 8. Table 3 summarized their effects, if any, on peak integrated areas, this calculated by a method that considers area between the apex and a horizontal line drawn at 1/5^th ^the height of the apex. Looking at Figure 8, Peak #1 appears to have been least precisely aligned by XCMS, peak #4 by COW. For COW and XCMS, the less symmetric peaks #2 and #3 appear to show some dependence of apical position on the height of peaks, a phenomenon not evident with block-shifting. Table 3 illustrates that COW, and to a lesser extent, XCMS alignments are accompanied by artifactual distortions in peak area. We also observed peak *shape *differences (data not shown). As for the block-shift method, areas of two of the four peaks were perfectly preserved. Two and 13 of 45 analytical runs did show area distortion for peaks #2 and #3, respectively. This can be attributed to the inclusion of a peak tail region during integration which was excluded from the peak region during block-shift alignment, thus, was liable to be warped.

**Table 3:** Peak integration errors* caused by three alignment methods

	**1**	**2**	**3**	**4**
**COW area %error ± SD**	8.7 ± 5.2	4.7 ± 3.8	3.0 ± 2.4	4.5 ± 3.2
**XCMS area %error ± SD**	0.17 ± 00.14	1.29 ± 0.91	0.50 ± 0.89	0.11 ± 0.10
**Block-shift area %error ± SD**	0.000 ± 0.00	0.002 ± 0.01	0.18 ± 0.80	0.000 ± 0.00

**Block *vs*. COW (*t*-test P val.)**	<10^-10^	<10^-10^	<10^-10^	<10^-10^
**Block *vs*. XCMS (*t*-test P val.)**	<10^-10^	<10^-10^	0.08	<10^-10^

*area %error = 100% × (area_aligned _- area_raw_)/area_raw_

## Discussion

Robust alignment is an important step as it affects not only the quality of comparative post-data analysis but also which type of data analysis can be used [9]. Our iterative block-shifting approach is well suited to subsequent data analysis methods that operate on matrices, because the discrete nature of the time axis is preserved, and should allow approaches that require trilinearity because resulting alignments are precise to within one scan unit. Additionally, it preserves areas and shapes of detected peaks.

Precise alignment is possible through the recurrent use of mass spectral information in both peak detection and peak matching steps. Some alignment errors may not be prevented by spectral considerations, however, for instance, errors that might occur when multiple isobaric compounds are retained differently during chromatography. There is an additional requirement for peak matching that the match not adversely affect the alignment of too many of the remaining peaks in its block (as set by the *prof *parameter). The effect of the *prof *criterion is to delay the matching of potentially troublesome peaks such as isobaric compounds, ultimately until they exist alone in a block, at which time, the desireability of using them for alignment is evaluated by a loess-based smoothing criterion. This method potentially can calibrate even heavily misaligned peaks since peaks are found in an adjustable search range; we know of no other alignment algorithm for which the deviation in retention time from sample to sample can exceed the time between a peak and its neighbors [8,18].

One drawback of iterative block shifting is that, while its final step of warping and shifting conserves detected peaks, undetected peaks are liable to be deformed since nonpeak regions are warped. For this reason, the stringency during detection of sample TIC peaks is kept very low to try to detect, and thus preserve, most or all peaks of experimental interest. In cases where much of the retention artifact occurs at the beginning of the chromatogram, warping artifacts will be minimal, since the leftmost correction is a simple block-shift. Finally, if an undetected, and thus, potentially distorted peak is detected by some other means subsequent to alignment and proves important in the experiment, an investigator can always recover true peak area and shape by referring to the original raw data file using adjacent matched and aligned peaks to help locate the feature of interest. Because most peaks are area-preserved by this method, it is expected that fewer instances will occur than with methods that generally distort area that will require a return to the raw data for quantification purposes.

One disadvantage of typical pairwise alignment approaches is that the selection of the reference chromatogram can affect performance [9,26]. No sample chromatogram is likely to include every peak from all the other chromatograms in a series. Our proposed method, while not free from this disadvantage, lessens the difficulty of selecting a good reference by using subsets of available peaks in the reference for the alignment of every other sample.

## Competing interests

The authors declare that they have no competing interests.

## Authors' contributions

MC conceived, developed the algorithm, and drafted the manuscript. RJSR and JT coordinated the project and revised the manuscript. All authors read and approved the final manuscript.

## Supplementary Material

Additional file 1 Alignment result if run 1 is selected as the Reference. Click here for file

Additional file 2 Alignment result if run 22 is selected as the Reference. Click here for file

Additional file 3 Alignment result if run 36 is selected as the Reference. Click here for file
