# Supplementary figures and images for: An iterative block-shifting approach to retention time alignment that preserves the shape and area of gas chromatography-mass spectrometry peaks
Source: BMC Bioinformatics. 2008 Aug 12;9(Suppl 9):S15. doi: 10.1186/1471-2105-9-S9-S15 (PMC2537566; doi:10.1186/1471-2105-9-S9-S15)

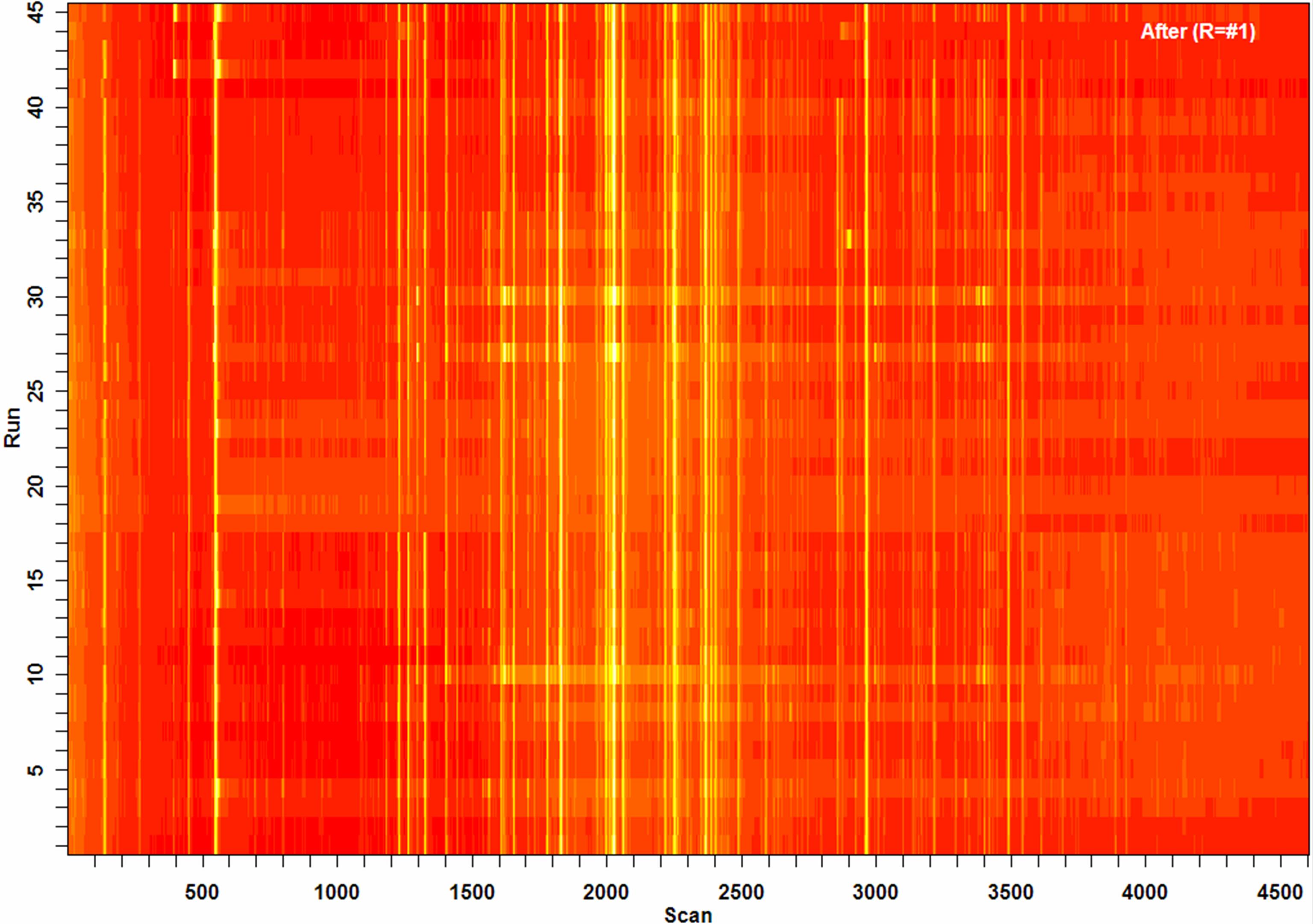

Supplement: Additional file 1 — Alignment result if run 1 is selected as the Reference. [file 1471-2105-9-S9-S15-S1.jpg]

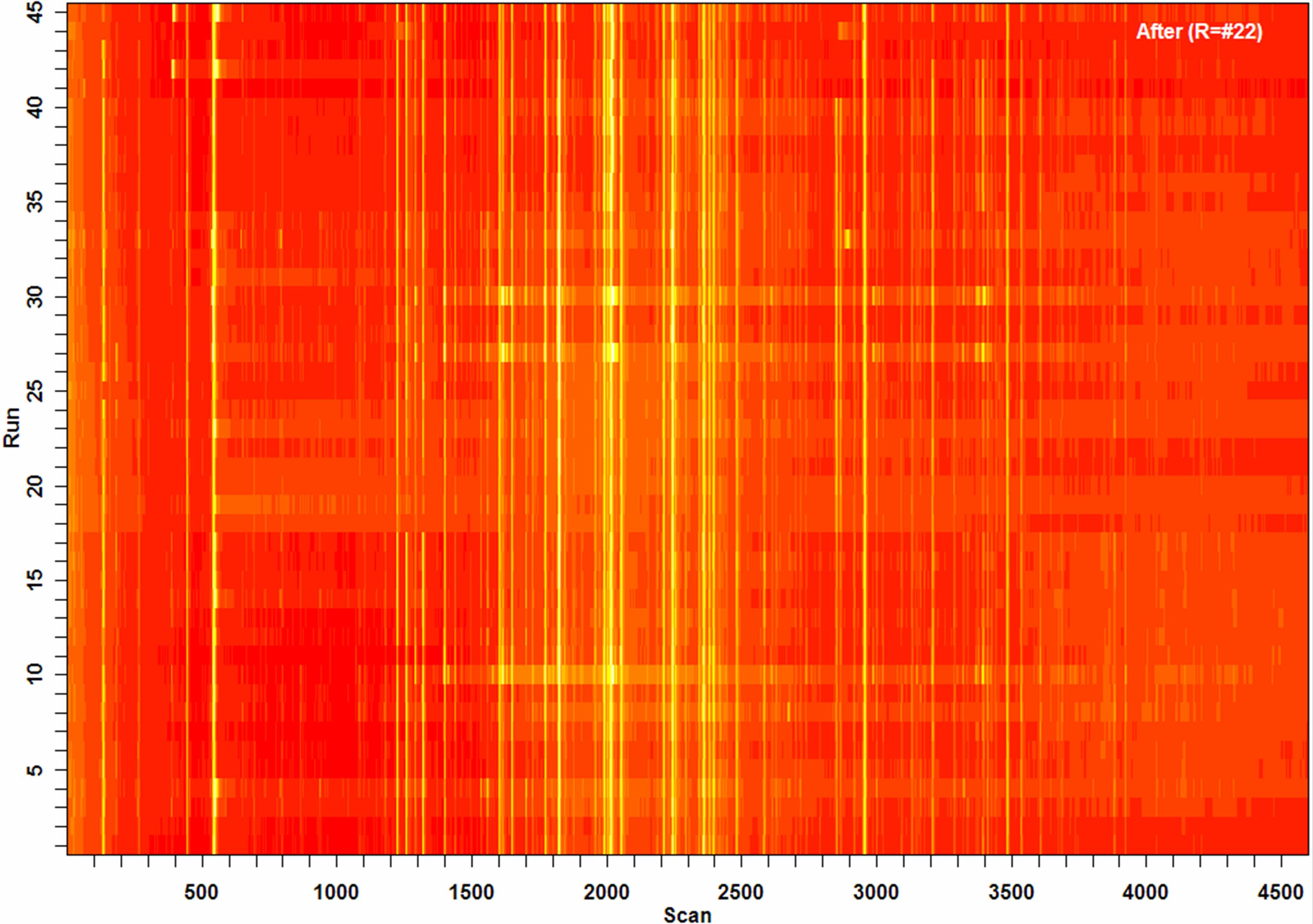

Supplement: Additional file 2 — Alignment result if run 22 is selected as the Reference. [file 1471-2105-9-S9-S15-S2.jpg]

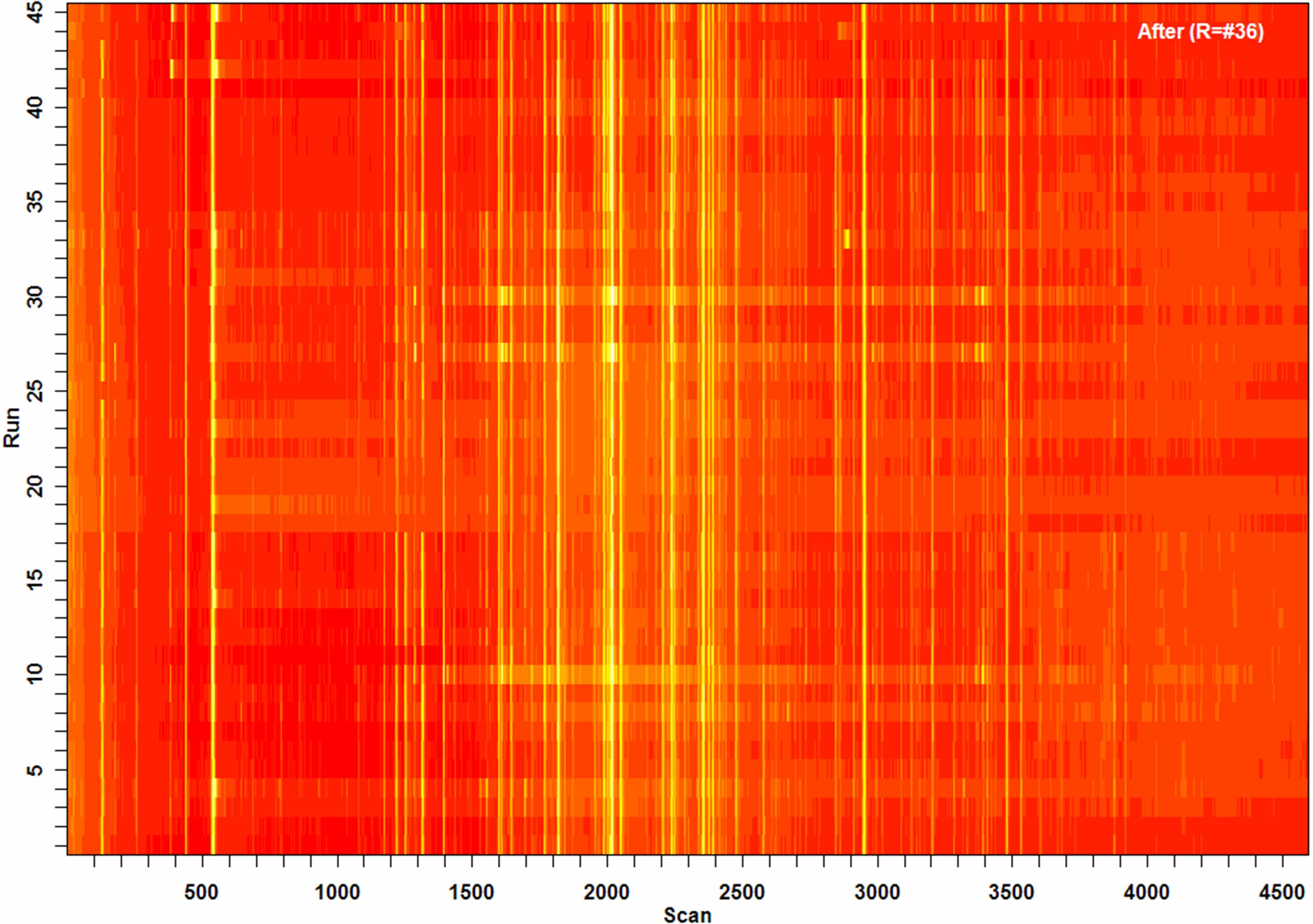

Supplement: Additional file 3 — Alignment result if run 36 is selected as the Reference. [file 1471-2105-9-S9-S15-S3.jpg]
